# Correction

**DOI:** 10.1111/cas.15534

**Published:** 2022-10-04

In an article^1^ titled “miR‐124‐3p regulates cell proliferation, invasion, apoptosis and bioenergetics by targeting PIM1 in astrocytoma” by Danni Deng, Lei Wang, Yao Chen, Bowen Li, Lian Xue, Naiyuan Shao; Qiang Wang, Xiwei Xia, Yilin Yang, and Feng Zhi, the following errors were published:

In Figure 5, Figure 5F have been replaced, the correct figure is presented below. 
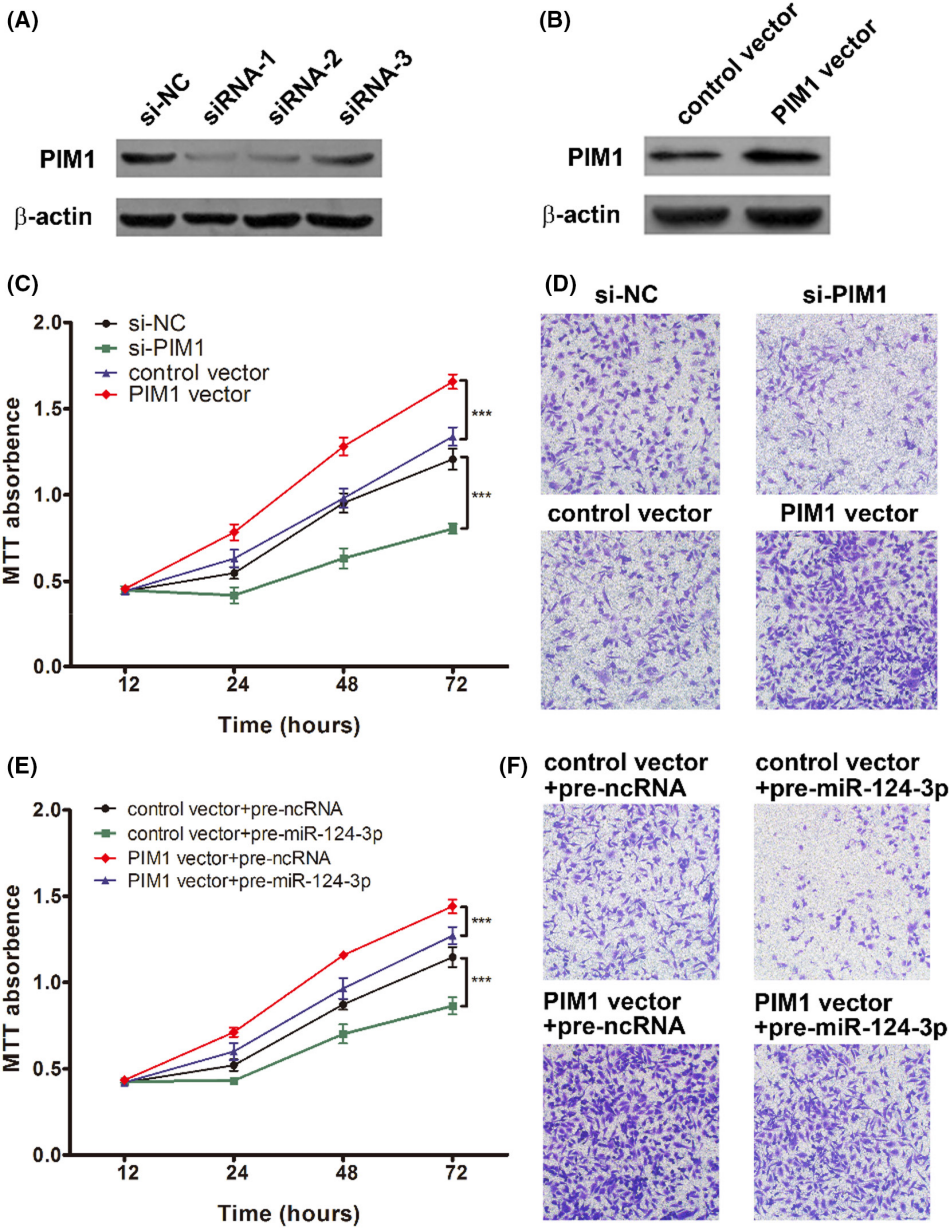


The authors apologize for the error.

## References

[1] Deng D , Wang L , Chen Y , et al. miR‐124‐3p regulates cell proliferation, invasion, apoptosis and bioenergetics by targeting PIM1 in astrocytoma. Cancer Sci. 2016;107:899‐907. doi:10.1111/cas.12946 27088547 PMC4946703

